# Tidal and diel orchestration of behaviour and gene expression in an intertidal mollusc

**DOI:** 10.1038/s41598-018-23167-y

**Published:** 2018-03-20

**Authors:** Y. Schnytzer, N. Simon-Blecher, J. Li, H. Waldman Ben-Asher, M. Salmon-Divon, Y. Achituv, M. E. Hughes, O. Levy

**Affiliations:** 1 The Mina and Everard Goodman Faculty of Life Sciences, Bar-Ilan University, Ramat-Gan, Israel; 2 Present Address: Eugene Bell Center for Regenerative Biology and Tissue Engineering, Marine Biological Laboratory, Woods Hole, MA USA; 3 Division of Pulmonary and Critical Care Medicine, Washington University School of Medicine, St. Louis, MO USA; 4 Department of Molecular Biology, Ariel University, Ariel, Israel

**Keywords:** Transcriptomics, Transcriptomics, Animal behaviour, Animal behaviour

## Abstract

Intertidal inhabitants are exposed to the 24-hour solar day, and the 12.4 hour rising and falling of the tides. One or both of these cycles govern intertidal organisms’ behaviour and physiology, yet little is known about the molecular clockworks of tidal rhythmicity. Here, we show that the limpet *Cellana rota* exhibits robust tidally rhythmic behaviour and gene expression. We assembled a *de-novo* transcriptome, identifying novel tidal, along with known circadian clock genes. Surprisingly, most of the putative circadian clock genes, lack a typical rhythmicity. We identified numerous tidally rhythmic genes and pathways commonly associated with the circadian clock. We show that not only is the behaviour of an intertidal organism in tune with the tides, but so too are many of its genes and pathways. These findings highlight the plasticity of biological timekeeping in nature, strengthening the growing notion that the role of ‘canonical’ circadian clock genes may be more fluid than previously thought, as exhibited in an organism which has evolved in an environment where tidal oscillations are the dominant driving force.

## Introduction

Life on earth has evolved under constant environmental change. The daily, tidal, lunar and annual cycles are the most significant, affecting different life forms in their respective habitats. Consequently, organisms time their biochemical, physiological, and behavioural processes in the most advantageous manner, in phase with the predictable environmental change^1^. Indeed, orchestration of the endogenous clock with the environment enhances the organism’s fitness and survival^2,3^. Almost all organisms, from bacteria to humans, have evolved an endogenous clock with a periodicity around 24 hours^4^, driving high levels of cyclical gene expression^5^. The architecture of circadian clock networks arise from the interactions between two transcriptional/translational feedback loops comprised of positive and negative elements^6^. However, there are differences in the specific clock components and their interactions between taxa^2^.

The intertidal zone is an exceedingly complex habitat subject to a multitude of cyclic environmental stimuli^7^. The predictable cycle of inundation and exposure brings about rapid changes in temperature, salinity, hydrostatic pressure, food, and predation pressure^8^. Therefore, tidally-timed endogenous rhythms have evolved in most intertidal organisms^9^. As with the circadian clock, these endogenous rhythms persist for some time under constant conditions without tidal cues, can be entrained by appropriate stimuli, and are temperature compensated^10^. However, very little is known about the underlying molecular clockworks of tidal rhythms. Studies on intertidal animals have shown that putative circadian clock genes are not always expressed in a typical circadian pattern^10–12^. Furthermore, applying RNAi silencing on them does not affect circatidal rhythmicity^10,13,14^. In contrast, inhibition of a known circadian clock gene, *Casein kinase I*, lengthens the circatidal rhythm of swimming behaviour in *E*. *pulchra*^10,15^. Surprisingly, the presence of an independent 12 hour clock has recently been identified in mammalian cells, suggesting a possible evolutionary remnant of an ancient tidal clock^16^. Thus, progress is being made toward the characterization of the molecular underpinnings of tidal rhythmicity; however, this field is still in its infancy.

Here we explored the rhythmic behaviour, at sea and in the laboratory, in conjuncture with temporal gene expression profiles of a key intertidal mollusc, the Red-Sea limpet *Cellana rota*^17–20^. From an evolutionary perspective, an understanding of tidal clocks is of much importance to the general field of chronobiology. The ‘tidal clock’ governing the rhythms of intertidal organisms is manifested in some of the most ancient animals. Clock mechanisms would have developed before the divergence of the major animal clades, existing in a common ancestor, occupying bodies of water in which tidal cycles would have been as ecologically important, if not more so than the circadian cycle^21,22^.

## Results and Discussion

### *C*. *rota* activity in the field

**Figure 1:**
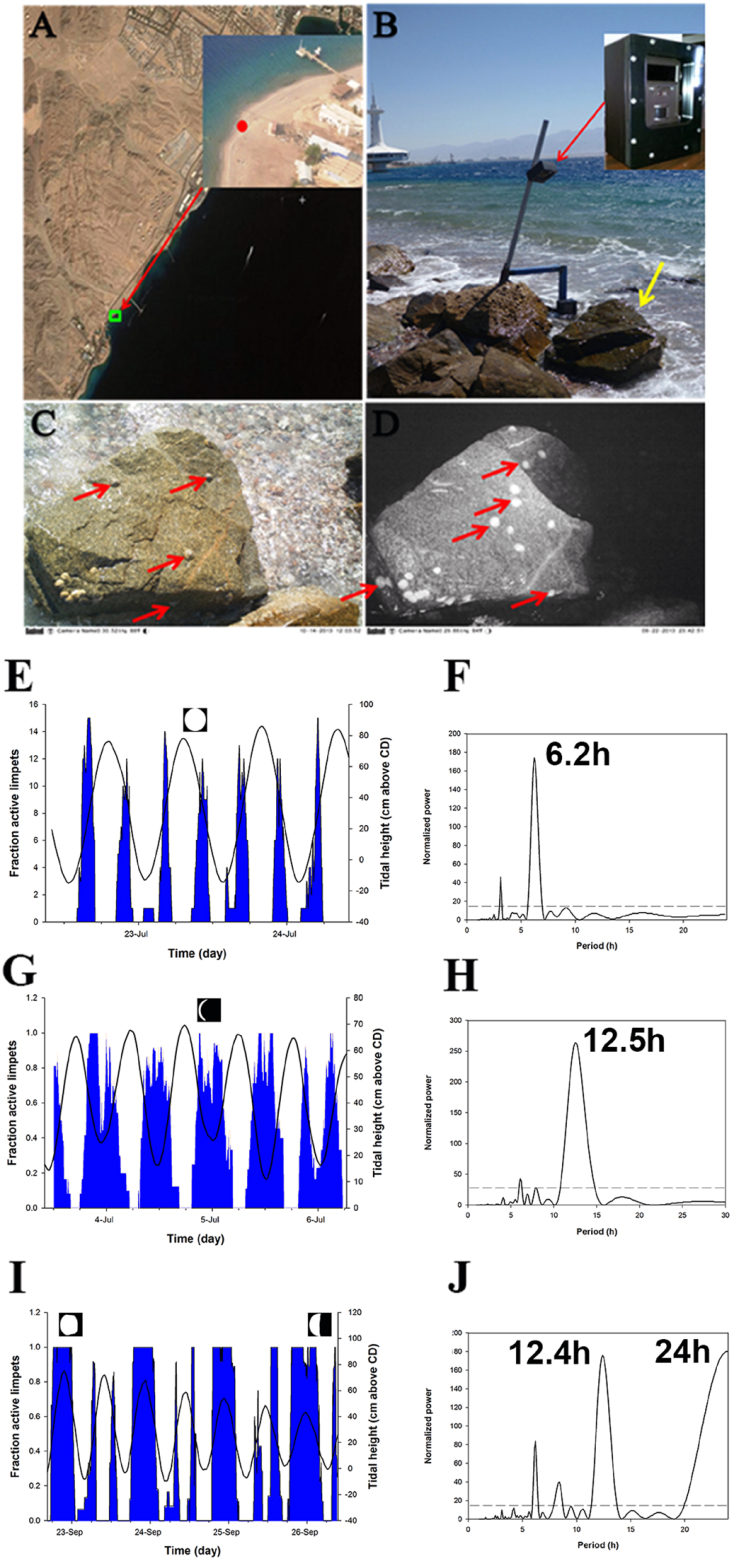
*Cellana rota* behaviour in the field. (**A**) Location of observation site. The background image was created by screen-shot from Google Earth (v7.1.8.3036, https://www.google.com/earth/), and the source image was provided by ©2017 CNES/Airbus Image DigitalGlobe. (**B**) Trail camera (Trophycam HD, Bushnell Co.) placed in a custom made waterproof housing mounted on a stable pole fixed to a boulder. The camera is pointing at the target boulder (marked with yellow arrow) monitoring a natural population of *Cellana rota*. (**C**) Image of target boulder during the day. (**D**) Image of target boulder with IR illumination at night. Sample of *C*. *rota* individuals marked with red arrows. (**E**–**J**) Typical locomotor modalities (left) and LSP periodograms (right; p > 0.05). (**E**) Bimodal periodicity 3/7/2013–6/7/2013 (n = 18), (**G**) Unimodal periodicity 22/7/2013–24/7/2013 (n = 18), (**I**) Diel periodicity 22/9/2013–26/9/2013 (n = 17). The blue filled plot represents the fraction of active limpets. The black line plot represents the sea level at the time. X-axis is time (days), primary y-axis is fraction of active limpets, and secondary y-axis is sea level. Moon phase is presented above the graph. Numbers of limpets monitored during each time period are noted above (max observed during given period). LSP periodograms (**F**,**H** and **J**) are presented to the right of each corresponding activity graph. Most significant peaks are labeled (p < 0.05). Dashed line in the periodograms indicated the 95% confidence intervals.

In order to establish the limpet’s rhythmicity under natural conditions we monitored a boulder with a population of *C*. *rota* over an extensive period of time (initially 15–20 individuals, then up to 100 after further settlement; See limpet identification in materials and methods). Time-lapse images of the boulder were taken every 5 min’s, during the day and night. *C*. *rota* exhibited significant tidal rhythmicity throughout all the periods analyzed (Figs 1 and S1; Table S1; Movie S1). Previous studies have shown that limpets have either a circatidal or mixed circatidal/circadian rhythm^23,24^. *C*. *rota* exhibited three locomotor modalities. During most periods there was a clear bimodal tidal periodicity; the first activity bout commencing with the rising tide until immersion, at which point they cease movement. The second bout began with the falling of the tide and continued until the boulder began to dry (Figs 1E and F and S1B–D,F,H, and I). During other periods there was a distinct unimodal pattern, whereby the limpets were active between the falling of the tide, throughout most or all of the low tide, and continuing with the rising tide until immersion (Figs 1G and H and S1E and J). As the sea level moves from neap (low amplitude) to spring (high amplitude) tide there is a clear shift in behaviour (Fig. S1A and I). During the third modality, observed only in September and October of 2013, the limpets exhibited tidal rhythmicity with an additional diel component (Figs 1I and J and S1G). During these periods *C*. *rota* was active at night from the rising of the tide, throughout the entire high tide (immersed) and then ceasing movement as the boulder dried. Activity bouts were significantly longer at night than during the day in September 2013 (Mann-Whitney U test; U = 0, n = 7,4 p = 0.006). Why *C*. *rota* adopts this diel modality for a short period of time is unclear. Hulings^19^ reported similar findings during the same period of the year. A possible explanation is a gradual rise in sea level which occurs during September^19^. The change in sea level may be a cue for some other behaviour, such as breeding, which occurs during the later summer - early winter months^20^. Indeed, limpets modulate their optimal timing of activity according to specific local abiotic and biotic factors^25,26^. Moon phase significantly affects *C*. *rota* rhythmicity (nested ANOVA; *p* < 0.0001, *F* (5, 8706) = 111.14), the highest proportion of activity being during the first quarter (0.73 ± 0.30; Tukey post-hoc test; p > 0.01 for all comparisons), and the lowest during the full moon (0.32 ± 0.39). Season has an impact on limpet locomotion (nested ANOVA; *p* < 0.0001, *F* (*3*, *8731*)* = *103.04), with a higher proportion of activity during the winter (Tukey post-hoc test; Winter > Autumn > Summer > Spring; p < 0.0001 for all comparisons). Similar seasonal patterns of activity have been observed in other limpet species^23^. Given Eilat’s arid climate, with high temperatures and desiccation stress in the spring and summer, this behaviour pattern seems favorable. Thus *C*. *rota* adopts a range of temporally distinct rhythmic patterns in its natural environment, revealing a complex and sophisticated plasticity of rhythmic behavior which takes into consideration a range of short and long term rhythmic environmental variables.

### *C*. *rota* activity in the laboratory

**Figure 2:**
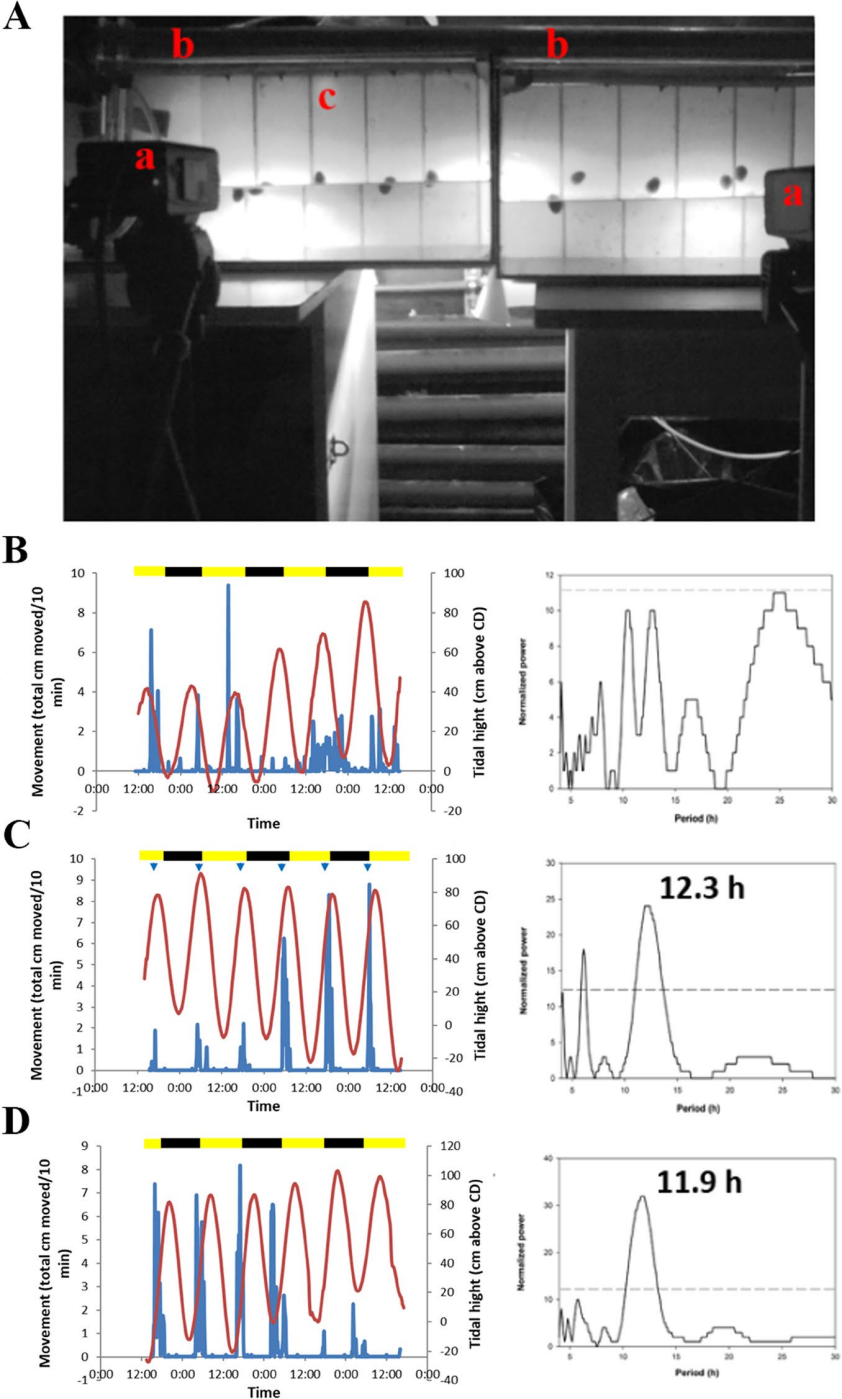
*Cellana rota* behaviour in the laboratory (**A**) Laboratory setup. (a) Cameras connected to PC with Ethovision tracking software. (b) Experimental Aquaria. On top is the fluorescent light providing ambient light per trial. Below that is a row of drippers providing water spray. Each aquarium is divided into five arenas, with individual limpets on back vertical wall, separated by glass barriers. (c) Example of arena. Dripper on top, below it is the lane with one *C*. *rota*. Behind the aquaria are the IR lamps providing backlighting for the tracking system. Each aquarium is 1/3 filled with water. (**B**–**D**) Typical recording of a *C*. *rota* individual’s locomotion under three consecutive laboratory conditions. (**B**) Arrhythmic limpet (2 weeks post collection) free run under DD and constant spray (69.5 hours; τ = N.S). (**C**) Entrainment to tidal cue (66.5 hours; τ = 12.3 h). (**D**) Free run (DD, no tide; 98 hours; τ = 11.9 h). The primary y-axis represents limpet movement, plotted in blue. The red line plot represents sea level at the collection location during the time of the experiments (secondary y-axis) and the X-axis represents time. Arrowheads denote time of spray turning on for a period of 3 hours each time (spaced 12.4 hours apart). The yellow and black bars represent the natural light phase at the time of the experiment which was conducted under DD conditions. LSP periodograms (right; p > 0.05).

Using computer-based tracking we were able to document the movement of *C*. *rota* in the laboratory (Fig. 2A). Limpets collected directly from the sea and placed under constant and varying light conditions maintained a significant circatidal rhythm (~12.4 h) for at least several cycles (Fig. 2; Table 1, Exp. 1–3). A previous study showed that *C*. *radiata* (junior synonym of *C*. *rota*) has an endogenous circatidal rhythm of oxygen consumption^16^. A second significant ~8 hour activity peak was also present in some of the limpets (Table 1. Exp. 2; Fig. S5), with a further fraction being arrhythmic. Circatidal rhythms are known to be notoriously noisy and often mixed with other apparently non-environmental periodicities^22,27,28^. Interestingly, a recent study on mammalian cells found robust 24, as well as 12 and 8 hour rhythms with potential biological significance^16^. From an evolutionary perspective there is presumably an adaptive advantage to a wide-ranging repertoire of ‘circa’ periodicities, particularly when residing in a complex environment such as the intertidal zone. After establishing their rhythm immediately post collection and ruling out the effect of light phase as an entraining cue, concurring with the majority of our observations in nature, experiments were conducted in order to entrain the limpets to a simulated tidal spray (Table 1, Exp. 2–4). The limpets were maintained under constant spray and light conditions (DD) for 2 weeks prior to entrainment in an attempt to desynchronize their rhythm. As expected, during the initial constant phase, most of the limpets exhibited no significant rhythmicity (Fig. 2B). Interestingly, some still maintained significant circatidal rhythmicity even after a month in seclusion from any apparent tidal cues. Once starting the spray entrainment regime, the limpets regained significant circatidal rhythmicity, with several remaining arrhythmic (Fig. 2C). Finally, the limpets, which were previously entrained, were monitored once again under constant spray and light conditions. They exhibited a significant circatidal periodicity, with some remaining arrhythmic (Fig. 2D; Table 1, Exp. 4). Taken together with the field observations, this series of experiments indicate that *C*. *rota* has a *bona fide* endogenous circatidal clock.

**Table 1:** Trials conducted under experimental conditions in the laboratory.

Experiment No.	Limpets (n)	Duration (hr)	Time from collection	Light	Spray	Period (τ)
1	5	147.5	DFS	DD	Constant	12.1 ± 1.1 (n = 5)
2	10	72	DFS	LD	Constant	12.16 ± 0.45 (n = 4) 8.07 ± 0.30 (n = 5) arrhythmic (n = 1)
	10	85		LD	Tide	12.09 ± 0.44 (n = 9)
						arrhythmic (n = 1)
	10	82		LD	Constant	NA
3	5	69.5	DFS	LL	Constant	12.88 ± 1.45 (n = 5)
	5	66.5		LL	Tide	12.75 ± 0.91 (n = 5)
	5	98		LL	Constant	12.36 ± 1.2 (n = 5)
4	20	76	2WL	DD	Constant	11.8 ± 0.45 (n = 5)
						arrhythmic (n = 15)
	19	71.5		DD	Tide	12.25 ± 0.40 (n = 14) arrhythmic (n = 5)
	13	73		DD	Constant	12.26 ± 0.54 (n = 9) arrhythmic (n = 4)

Time from collection – DFS – direct from sea, 2WL – two weeks in lab under constant conditions before experiment; Spray – Constant – no tidal cue, Tide – spray timed to entrain; Period (τ) of locomotor movement calculated by Lomb-Scargle periodogram (Refinetti, 2007) presented as average ± S.D. Trial 2 entrainment (constant phase) missing.

### *C*. *rota* rhythm of gene expression

**Figure 3:**
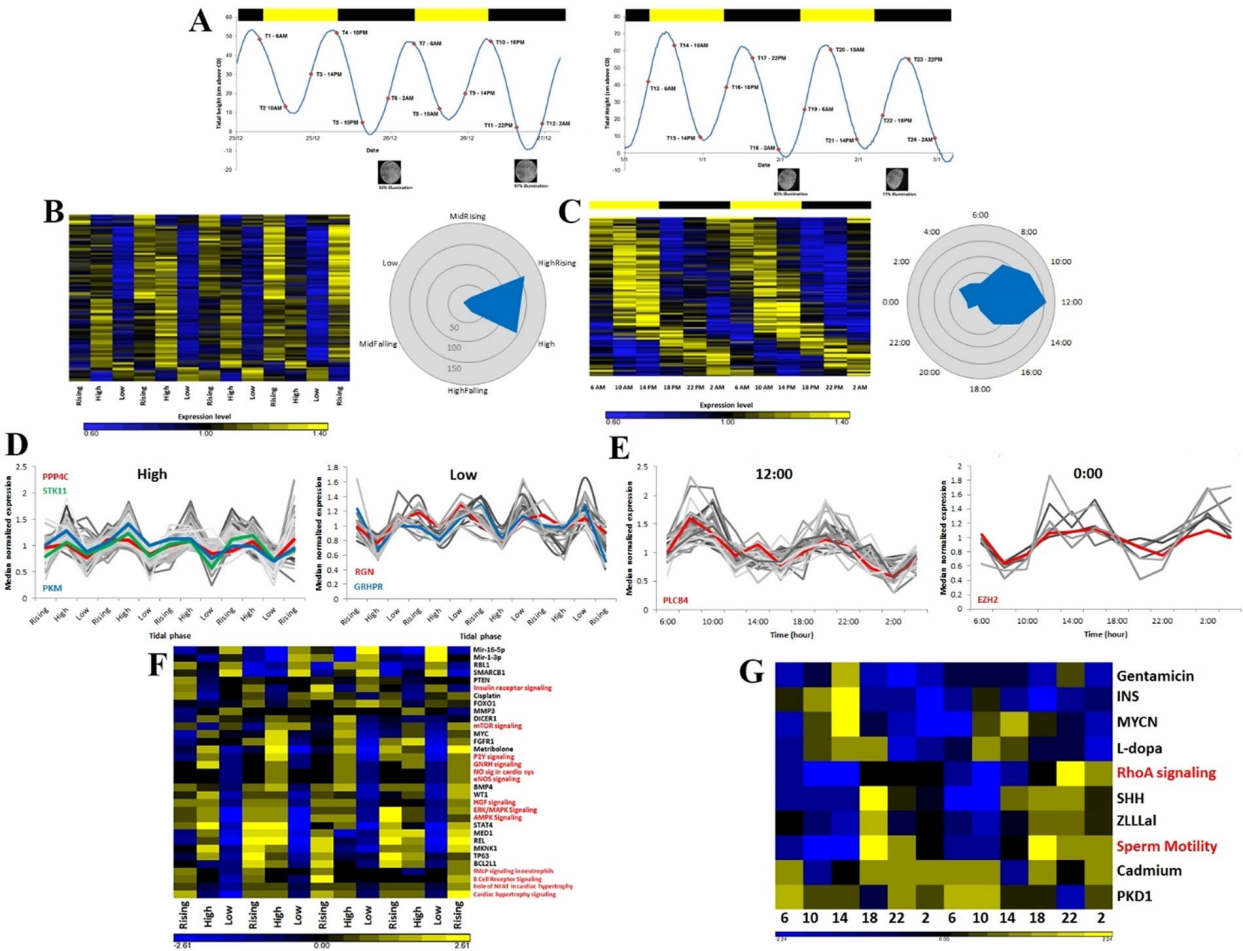
Transcriptome sampling design and analysis. (**A**) Sampling1 starting at 06:00 on the 25/12/2012 and ended at 02:00 on the 27/12/2012 (left graph), Sampling 2 starting at 06:00 on the 1/1/2013 and ended at 02:00 on the 3/1/2013 (right graph). The tidal phase (sea level) for each sampling point is shown in the graphs. Y-axis is the sea level in cm above chart datum, x-axis is date and time. Black bars denote night, yellow bars are day. Moon phase and illumination are shown at the bottom of each graph. (**B**,**C**) Heat maps of 367 significant tidal (left) and 221 diel (right) cycling transcripts. Phase maps of tidal and diel transcripts (p < 0.005) to right of each heatmap. Phases are sorted by the phase values given by JTK_Cycle. Yellow and Back bars denote light and dark phases respectively (graph C). (**D**) Histograms of cyclers at high and low tide. (**E**) Histograms of cyclers at midday and midnight; High –low tide comparison (χ^2^ = 81.2, df = 1, p < 0.0001); Midday-Midnight comparison (χ^2^ = 21.4, df = 1, p < 0.0001). Representative circadian clock related genes marked in red or green, hypoxia/anoxia related in blue. (**F**,**G**) IPA pathway analysis for tidal (**F**) and diel (**G**) transcripts. Enriched pathways marked in red, predicted upstream regulators marked in black.

We present the first transcriptomic investigation of a mobile intertidal organism’s rhythmicity (SRP078773). To date, there have only been two previous studies of this nature, both conducted on sessile intertidal inhabitants, the mussel *Mytilus californianus*^11^, and the Oyster *Crassostrea gigas*^29^. To explore the rhythmicity of gene expression in *C*. *rota*, we assembled a *de novo* transcriptome using samples collected every 4 hours over the course of 2 days during 2 lunar phases (12 time points per sampling; Fig. 3A). Employing JTK_Cycle^30,31^ we identified 367 tidal (P < 0.005; Figs 3B and S2; Table S2; File S1), and 221 diel significant cyclers (Figs 3C and S3; Table S3; File S2). Significantly more transcripts peak during rising and high tides compared to low tide (Fig. 3B and D; χ^2^ = 81.2, df = 1, p = 2.2e-16). There are significantly more cycling genes during midday compared to night (Fig. 3C and E; χ^2^ = 21.4, df = 1, p < 0.0001). These findings are in good agreement with *C*. *rota*’s dominant tidal rhythmicity in nature and the laboratory. Furthermore, our 4 hour sampling resolution provides more statistical power to 24-hour over 12-hour genes^32^, thereby reinforcing our result that there are many more tidal than diel cyclers. In stark contrast, *M*. *californianus* was found to exhibit ~10 times more circadian than tidally oscillating transcripts^11^. Invariably, the fundamental differences between mobile and sessile organisms inhabiting the tidal zone will contribute to this major difference. There is presumably a far greater adaptive value to having an accurate circatidal timepiece in mobile intertidal animals who can relocate and chose when and where to be active^8^. A fundamental and unresolved issue is whether circadian and circatidal rhythms are governed by the same, shared, or unrelated mechanisms^33,34^. We identified 12 transcripts with a significant tidal rhythmicity, which are related to the circadian clock mechanism at varying levels of importance, as well as several others involved in anaerobic metabolism, hypoxia/oxidative stress, biomineralization and calcium signaling (Table S4). Among them, we identified one putative circadian clock gene, P*rotein phosphatse 4c*, which is involved in circadian period length (Fig. 3D)^35^. *COP9 signalosome complex subunit 8* (*CSN8*) was one of the most robust tidal cyclers in our analysis. The *COP9* signalosome has been implicated in the regulation of the *Neurospora*^36^, *Arabidopsis*^37^ and *Drosophila*^38^ circadian clocks. As these organisms have fundamentally different circadian clock mechanisms and genes, it is intriguing that this particular gene came up as tidally rhythmic. Another circadian gene of interest with significant tidal expression is *Btb domain containing 9*, which has been implicated in sleep disturbance and temperature sensing^39^. As temperature is a known entraining cue of tidal rhythms this is of particular interest. Two further tidally rhythmic genes of interest are *Tetraspanin5* and *Fbxl4* which are both involved in *Rhodopsin* homeostasis^40^. Rhodopsin has been implicated in moonlight detection^41^. Interestingly, *C*. *rota Rhodopsin* peaked around the middle of the night during the near full moon sampling we conducted. qPCR and ddPCR validations (Fig. S4; Table S5) exhibited a strong correlation between the qPCR/ddPCR and RNA-seq data based on log2 fold change measured between six different time points spanning both sampling weeks.

We identified a majority of the known circadian core clock genes. *Cry1* (P > 0.07), *Clock* (P > 0.07) and *Bmal1* (P > 0.01) exhibited moderate to weak circadian rhythms not upholding significance. The other circadian core clock genes identified exhibit neither diel nor tidal rhythms. These findings are not unusual, as other tidal animals appear to lack typical circadian clock gene expression^42^. IPA pathway enrichment analysis revealed a wide range of tidally enriched processes and potential upstream regulators (Fig. 3F; File S3), whereas only two enriched pathways were identified in the diel analysis (RhoA signaling and sperm motility; Fig. 3G; File S3). The tidal analysis revealed several noteworthy processes, among them, the mTOR pathway, a central regulator of cell metabolism^43^, known to be involved in hypoxia signaling^44^, photic regulation and entrainment of the circadian clock^45,46^. Hypoxia related pathways are of evident importance to tidal organisms, which are constantly exposed to dramatic changes in oxygen availability. AMPK and ERK/MAPK signaling too are involved in both hypoxia^47^ and circadian clock^48–50^ regulation. Finally, two Nitric Oxide related pathways, eNOS signaling and NO signaling in the cardiovascular system, both known to be regulated by the circadian clock^51^, are also tidally rhythmic. The most noteworthy upstream regulator identified was *myc*, which activates negative regulators of the heterodimeric BMAL1/CLOCK complex^52^. Interestingly, a *C*. *rota* homolog of *myc* is a significant tidal cycler (P < 0.003). Taken together these findings suggests that not only is the behavioural output of tidal animals in tune with their rhythmic habitat, but that a significant proportion of the underlying molecular mechanisms and pathways either regulate or are regulated by a yet to be characterized tidal timepiece which presumably shares common parts with the circadian clock.

At the micro-scale we greatly expand what is known about *C*. *rota*, a key intertidal species in the Red-Sea of which curiously little has been known until now. We present the first clock oriented transcriptome of a mobile intertidal organism, revealing many new potential leads, taking us a step further in the identification and characterization of the ‘tidal clock’.

## Materials and Methods

### Animal identification

The Identification of the limpets was conducted based on a guide provided in ref.^20^. *C*. *rota* has a particular gill structure, differentiating it from other locally occurring limpet species. In *C*. *rota*, the gills surround the entire body except the head region, whereas in other species the gills commonly surround the entire circumference of the body. The identification was confirmed by Dr. Ya’akov Dafni in Eilat. Finally, the identity of a representative sample of the limpets was validated by DNA identification. A sampling of limpets was conducted from three locations along the Eilat shore – IUI, Marine Observatory and the north shore marina. See below for further details.

### Field observations

A population of *C*. *rota* was monitored over the course of the years 2012–2015. A boulder with a stable *C*. *rota* population was found on the border between the IUI marine laboratory and the Eilat marine observatory (Fig. 1A). In order to constantly monitor the limpet’s movements on the boulder we used a trail-camera (Trophy Cam HD; Buschnell co.; Fig. 1B). The camera was placed in a custom made waterproof housing. The housing was mounted on a galvanized iron pole which was welded into a boulder adjacent to the target one. The camera was positioned approximately 1 meter above the target boulder at a slight angle in order to maximize the field of view (Fig. 1B) and was set to take an image every 5 minutes during the day and night (Fig. 1C and D). At night the camera employs an IR flash (850 nm) which is invisible to the human eye. Previous studies have shown that limpets are not sensitive to IR wavelengths^53^. Our preliminary observations concurred with this as we did not notice any irregular behavior from limpets which were shone with an IR lamp of the same wavelength. The limpets were identifiable from both the day (Fig. 1C) and night (Fig. 1D) images. The acquired images were saved on a memory stick in the camera and downloaded periodically. The camera was powered by 12 AA batteries, enabling several weeks of image acquisition. In practice, periodical camera failures (presumably due to prolonged exposure to extreme temperatures) reduced the total time of camera activity. Thus, approx. 300 days of images were acquired at a 5 minute resolution, equaling some 100,000 images. In total, 33 entire days were quantified, during 10 periods of time. Table S1 presents a list of periods for which images were acquired and analyzed (further details below).

### Behavioural analysis – Movement

In order to assess and quantify the rhythmic behaviour of *C*. *rota* we gathered time-lapse images as described above. The movement of the limpets was quantified manually as the high levels of “noise” (changes in light, water spray, algal growth, other animal movement etc.) inhibited the use of a computer based program as was used in the lab (see below). Movement was defined as any observable relocation from one point to another, or rotational movement at a particular point (commonly observed limpet behaviour). Since it was not possible to observe the boulder in its entirety, and it was only monitored from one angle, the analysis was conducted at the population and not individual level. Quantification of this nature is most common in limpet studies^19,53–55^. Thus, we noted the total number of limpets, and those moving between one image to the next and calculated the fraction of active limpets. Individuals often moved down a side of the boulder and were “lost”, thus only the number of visible limpets out of the total observed at a given time was recorded. The angle of the camera gave coverage of over 60% of the boulder, hence we felt confident that what we observe reflects the total activity on the boulder. Over the course of the study visual inspections were conducted around the boulder, above and below the water, during day and night, in order to verify this. A total of 10 periods, at different stages of the tidal phase and year were analyzed (Table S1). After scoring the behavior further analyses were conducted (see Statistical analyses). Eilat sea level data was obtained from The IUI database (http://www.meteo-tech.co.il/eilat-yam/eilat_en.asp).

### Collection of Limpets - general

Living specimens of *Cellana rota* were collected from boulders in the intertidal zone in Eilat during 2012–2013. The limpets were collected from boulders in the vicinity of the one used for the behavioural observations. When at rest, *C*. *rota*, like other limpets adhere very strongly to the surface they are on (hence the English phrase – *as stuck as a limpet*). Thus, a thin knife was employed to dislodge them from the surface. Only visibly unharmed animals were collected. Preferentially the animals are collected when they are mobile, to limit the stress induced by removing them, yet this was not always possible when sampling was conducted at times when the limpets were at rest. Rao^56^ found that gonads are developed in specimens *of C*. *karachiensis* (=*C*. *rota*) at sizes larger than 10 mm. Thus, only adult specimens were collected. In addition, emphasis was put on collecting animals from the same height in relation to the water level, usually just at the water level. All limpets were collected under NPA guidelines (Permits no. 2012/38699 and 2014/40243).

### Collection of Limpets – Laboratory behavioural assays

Limpets collected for behavioural assays were placed in a small plastic container with a little sea water, then transported to Bar-Ilan University and placed in an aquarium. The container was rinsed and the water replaced several times during the course of the transport. Any limpets which were clearly dead or which did not adhere to the aquarium walls after reaching Bar-Ilan were discarded. Such collections were conducted numerous times over the course of the study, for each trial new limpets were collected. After finishing a series of behavioural trials the remaining limpets were returned to Eilat (in coordination with the NPA guidelines).

### Laboratory behavior experimental conditions – general setup

The limpets brought to the laboratory for behavioural assays were placed in 45 liter aquariums (50*30*35 cm) with a standard filtration setup. Artificial sea water was prepared from an instant mix (Red-Sea Co.) and maintained at the same salinity as in Eilat (40 ppm). Since the aquariums were not connected to an external reservoir, water changes were conducted every other day. Salinity, pH and other chemical water tests were conducted regularly and maintained at the recommended levels. Evaporation was compensated by a float connected to a purified water tap. Water and air temperature were maintained at stable levels throughout each trial, corresponding to those in Eilat for each collection period. Each aquarium was 1/3 filled with water. Preliminary observations and trials indicated that this was the optimal water level as the limpets spend the majority of time on the interface between the water and air or on the sprayed surface out of the water. The experimental setup was placed in a dark room with the windows and door covered in order to attain optimal control of the lighting conditions per experiment. Up to 15 limpets were held in each holding aquarium. Limpets held in the general holding aquarium were placed to adhere on the walls of the tank. Prior to the limpets’ collection algae was grown on the walls of the aquarium for grazing. The experimental tanks were identical to the holding ones but had in addition a slab of glass, matching the height and width of the aquarium, divided into five 10 centimeters wide, 50 centimeters long arenas (Fig. 2A). Thin glass barriers were used to define the arenas and prevent the limpets moving from one arena to the other. One limpet was placed in each arena, adhered to the wall of the glass slab. As with the holding tanks, algae were grown on the glass slabs prior to the experiments. Unsurprisingly, initial observations suggested that without algae for grazing the limpets would move very little. A timer controlled fluorescent lamp was placed above the aquarium and set according to the specific experimental conditions for each trial (LL – constant light, LD – 12:12 hour light dark, or DD – constant darkness). Typically, 2 adjacent aquariums were used for the experiments, allowing for a total of 10 individual limpets. Three 880 nm IR LED arrays (IR-56, Microlight infrared illuminators) were equally spaced behind the aquarium setup to provide illumination for the video tracking (details below). A sheet of white plastic was taped to back of each aquarium in order to maximize the diffusion of IR illumination around the arenas. As mentioned above, this wavelength is unobtrusive to the limpets.

Preliminary observations suggested that the limpets would move very little unless there was a constant spray of water on them. Previous studies in similar limpet species have indicated as much^53^. A spray system was devised to this end. A 350 Liter/hour power head pump (ViaAqua Co.) was placed in the front corner of each aquarium so as to not obstruct the view of the limpets. The pump was connected to a plastic tube which was ran along the side of the aquarium and then along the entire length of the tanks back wall (above where the limpets were placed). Five small garden drippers (Netafim Co.) with a 5-millimeter opening were connected to the main tube at equal spaces, each right above one of the five arenas. Consequently, it was possible to provide a constant spray of water on the limpets and keep the algae grown walls moist. Initial calibrations were conducted to optimize the water flow. This spray system was always on except for tidal entrainment experiments.

### Behavioral assays

The primary purpose of the behavioral assays was to establish whether the limpets possess an endogenous clock (circadian and/or circatidal). In the case of circadian rhythms, it is accepted that if animals are placed, upon collection, under constant lighting conditions (LL or DD) and they maintain their natural rhythm for at least several cycles, then they presumably have an endogenous clock that is timing and controlling their behavior. In the case of circatidal rhythms the same rules apply but with changes in water levels or other tidally associated cues [e.g. changes in water level, temperature, salinity, and hydrostatic pressure]^8^.

Limpets were collected from the shore in Eilat and brought to the experimental setup at Bar-Ilan University as fast as possible (~4 hours). The purpose of the initial experiments (Table 1; Exp. 1–3) was to establish how the limpets behave, particularly with regard to their rhythm of movement, during the time after they were removed from the sea. Experiment No. 2 was conducted under light conditions matching those in Eilat (LD) in order to test for light entrainment (circadian effect), and experiment 1 and 3 were held under constant spray and light conditions (DD or LL) to assess the limpets behaviour under constant conditions. In the final experiment (Table 1; Exp. 4) the limpets were brought to the lab and kept in holding tanks under constant conditions for 2 weeks under constant darkness (DD). Experiments 2–4 were composed of three stages:

1. Constant conditions (spray and light) – This stage was conducted in order to test for the limpets free run rhythm.
2. “Tidal” spray – the limpets were exposed to 3 days (6 tidal cycles) of spray timed to the Eilat high tide at the time. Tidal prediction data was downloaded from www.tides4fishing.com. Each high tide was approximately 12.4 hours apart, and with a daily shift forward of ~50 minutes. The spray was timed to turn on 1.5 hours before high tide, and turn off 1.5 hours after high tide, mimicking the average time spent by the limpets awash during a typical tidal cycle (corroborated with field observations). The purpose of this stage was to see if the limpets would entrain to the artificial rhythmic cue provided.
3. Constant conditions (spray and light) – This stage was aimed at seeing whether the limpets indeed became entrained to the previous stages manipulation.

Upon completion of each trial the limpets were returned to the holding tanks.

### Acquisition setup, data collection and analysis

We employed the video based tracking system Ethovision XT10 (Noldus Information Technology, Wageningen, The Netherlands) for the monitoring of limpet movement during the trials. Limpets were placed in an aquarium, as described above, and were tracked using Ethovision. The limpets were placed in the aquarium at least 2 hours before the start of a trial in order to acclimatize. Two Panasonic CCTV video cameras (WV-CL930/G) were positioned in front of the aquarium, one per tank (Fig. 2A). The cameras had a 4.5–12.5 mm lens (Computar, Japan), enabling an adequate field of view. The cameras were placed on tripods and secured in place. An 850 nm IR filter (RG850, Heliopan Gmbh, Germany) was fixed to the lens of each camera in order to enable tracking of the IR illuminated aquarium, and at the same time prevent the change in ambient lighting conditions interfere with the tracking. The cameras were connected to PC (Dell Precision T3500) and controlled via Ethovision. The detection setting of the program was set to detect moving black pixels. As the application of the Ethovision tracking system in limpets is novel, a series of calibrations was required in order to best utilize the setup for our purposes. Ethovision is commonly used for either fully dry or fully wet conditions. The application at hand, with changes in water level and constant spray, required some challenging adaptations. In order to calibrate the signal to noise ratio and detection settings of the program, immobile and mobile limpets were monitored^57^. To set the lowest threshold of activity for distance (measured in centimeters), the basal activity of 10 immobile limpets was measured for 10 minutes. The average values of “immobile” movement were defined as the lowest threshold of activity and concurred with visual observations conducted at the time. The program parameters for detection were set as follows: grayscale, range set between 1 and 100, subject size of minimum 1 pixel and maximum 8,000 pixels, contour erosion set at 1, contour dilation set at 5, pixel smoothing, and video sample rate of 1 frame per second. These parameters were tweaked for each particular trial as the difference in limpet size and algae background required slight alterations. After setting up the detection settings each trial was recorded. Typically, the program only records the tracking information and not the entire video recording due to the large volume of computer space required. For some of the trials video recording was also conducted in order to verify the tracking. Also, once recorded it is possible to re run the tracking under different parameters in cases where a limpet was ‘lost’ during the original experiment.

After the completion of a trial the raw tracking data was subjected to the programs filtering as follows: distance threshold set at 0.08 cm; time threshold, averaging interval of 1, start velocity of 0.1 cm/s, and stop velocity of 0.09 cm/s; nested movement calculation set for not moving. The data were converted into 10 min time bins for further analysis. Smoothing (lowess) was set based on 10 samples before and after every sample point. This step further insures the elimination of noise. In the event tracking was lost for small periods of time the interpolation function was implemented. These changes were verified on the video recordings when available. The data was then exported to Excel (Microsoft) for further analysis. The cleaned movement data was plotted against the time, sea level (at collection location; sea level data was downloaded from http://www.meteo-tech.co.il/eilat-yam/eilat_en.asp), light phase, and experimental spray cycle if altered. See statistical analyses for further details.

### Collection of Limpets – for Identification

One sampling was conducted in order to verify the identity of the limpets monitored/collected. Five limpets were collected at three sites along the Eilat shoreline, IUI – Marine laboratory, Eilat Observatory and the north shore marina (n = 15 in total). Random limpets were collected based on the identification process mentioned earlier. The limpets were placed in absolute alcohol, marked and transported to Bar-Ilan University for further analysis.

### DNA extraction, amplification, sequencing and analysis

DNA was extracted from the alcohol-preserved specimens using high pure PCR template kit (Roche; Germany). Dream Taq Green (#K1081, Thermo Scientific) was used for amplification by the polymerase-chain-reaction (PCR)^58^ with 50 ng DNA per reaction. COI was chosen as the representative gene used for identification. It was amplified using PCR with the universal primers LCO1490 and HCO2198^59^. The COI DNA was amplified by performing 40 cycles of 30 s at 94 C, 45 s at 47 C and 15 s at 72 C, followed by a final extension of 7 min at 72 C. PCR products were purified by centrifugation through a high pure PCR product purification kit (Roche Diagnostics GmbH, Mannheim, Germany). PCR products were sequenced on both strands by MCLAB (Molecular Cloning Laboratories, USA). Sequences were then subsequently manually inspected and edited using the BioEdit program^60^. After comparing the sequences and creating a consensus sequence, we employed the NCBI-BLAST utility to establish their identity. The sequences have been deposited to the GenBank database under the accessions: KX453271-KX453283.

### Limpet identification

The limpets collected for DNA identification from three different locations along the Eilat shoreline all exhibited identical partial COI sequences. A consensus sequence was created and blasted (NCBI-BLAST) against the NCBI database. The blast resulted in a 100% sequence identity (99% query coverage) to *C*. *eucosima* (a junior synonym of *C*. *rota* as mentioned earlier). The second blast hit was a *C*. *rota* sequence with 99% identity (91% query coverage). The sequences have been deposited to the GenBank database under the accessions: KX453271-KX453283.

This analysis provides strong proof that the limpets observed and collected during this study are indeed *C*. *rota*. It further establishes with a good degree of confidence that *C*. *rota* is currently the dominant limpet along the Eilat shoreline.

### Collection of Limpets – for transcriptome

We conducted one major sampling for RNA extraction and transcriptome sequencing (Fig. 3A). Individuals of *C*. *rota* were collected directly from the sea. For this purpose, an appropriate collection site was identified. An emphasis was placed on conducting the sampling at a site as close and as similar as possible to the one used for the behavioural observations. We chose two adjacent boulders with large *C*. *rota* populations (>200) opposite the IUI – Marine laboratory, approximately 100 meters south of the boulder used for monitoring behaviour. The limpets were sampled on two occasions, a week apart, at 4 hour intervals during two consecutive days (24 sampling points × 5 limpets = 120 samples). The first sampling started on the 25/12/2012 at 06:00 and ended on the 27/12/2012 at 02:00. The second sampling was conducted between the 1/1/2013 at 06:00 and the 3/1/2013 at 02:00. The weather during over the course of the collection was fairly constant, very partial cloud cover with no rain. The air temperature ranged between 17–20 degrees Celsius, and the sea temperature was constant around 23 degrees Celsius. The first week sampling was conducted during the waxing, three days right before a full moon (92–99% illumination), and during the second sampling the moon was waning (85–68% illumination). The rationale behind the sampling times was that the solar day length between one week to the next changes very little. However, the tidal phase shifts approximately 50 minutes forward every day. So, for example, a sampling during the first week at 10:00 would be high tide, whereas during the second week it would be low tide. Thus, by sampling at the same solar times during the two weeks we would be able to identify both tidal and diel rhythmic genes.

### RNA extraction

Total RNA was isolated from each of the collected limpets separately. Each limpet was removed from the freezer and placed on ice. Using a scalpel, each limpet was removed from its shell and head cut off right above the “shoulder” mark. In order to minimize the chances of contamination from consumed food the head was chosen for extraction. Also, preliminary extractions suggested this amount of tissue provides more than enough RNA for further use. The extraction was conducted with a Total RNA Mini kit (A&A Biotechnology, Poland) according to manufacturer’s instructions. Each RNA extraction was divided into three aliquots and frozen (−80 degrees Celsius). Electrophoresis on 1% agarose gel was run to check for RNA degradation. NanoDrop ND-1000 Spectrophotometer (NanoDrop Technologies, Wilmington, DE, USA) was used to estimate concentration as well as to obtain the purity A260/A280 ratio (>2.00). All the samples were then analyzed by Agilent 2100 Bioanalyzer (Agilent Technologies, Santa Clara, CA, USA) to confirm adequate RNA integrity. Commonly, RNA with a RIN > 9 is considered undamaged and adequate for further use. The RIN is calculated as a function of the 28 s/18 s rRNA ration. However, in the case of some protostomes, molluscs among them, the 28 s peak is not clearly visible or absent, resulting in a very low RIN number. The denaturative hydrogen bond breaks due to the extraction process, causing co-migration of the 18 S and 28 S bands, thus often only one clear peak is evident. Consequently, the 28 s/18 s rRNA ratio and RIN are not applicable^61–63^. So the readings were visually inspected based on the guidelines provided in Winnebek *et al*.^63^. All RNA samples exhibited a sharp 18 s peak, some with an additional small 28 s peak, and no additional signs of breakage were apparent.

### cDNA synthesis

cDNA was prepared from the individual RNA samples using a cDNA synthesis RevertAid First Strand cDNA Synthesis kit (#K1622, Thermoscientific). Oligo (dT)-primers (Invitrogen) were used for reverse transcription from 1000 microgram RNA per sample. cDNA was kept at −20 degrees Celsius.

### RNAseq – Library preparation and Transcriptome sequencing

For the transcriptome experiment we pooled 1 microgram of RNA from each limpet sample at a given time point (as described above). The samples were cold shipped to IGA Technology Services Srl in Udine, Italy where the sequencing was performed. The cDNA libraries were prepared at the sequencing site.

Upon arrival at the IGA total RNA of the samples was analyzed on Agilent 2100 Bioanalyzer system (Agilent, Waltham, MA) to evaluate that no damage occurred during shipment. The quality was assessed based on the same guidelines described above suited to limpet rRNA. Library preparation was conducted using ‘TruSeq mRNA Sample Prep kit’ (Illumina, San Diego, CA) according to the manufacturer’s instructions. Poly-A mRNA was fragmented 3 minutes at 94 °C and every purification step was performed using 1X Agencourt AMPure XP beads.

The final libraries were quantified by using a Qubit 2.0 Fluorometer (Invitrogen, Carlsbad, CA) and quality tested by Agilent 2100 Bioanalyzer High Sensitivity or DNA 1000 assay (Agilent Technologies, Santa Clara, CA). Libraries were then processed with Illumina cBot for cluster generation on the flowcell, following the manufacturer’s instructions and sequenced in paired-end mode on HiSeq. 2500 (Illumina, San Diego, CA), following the manufacturer’s instructions for a rapid run. The CASAVA 1.8.2 version of the Illumina pipeline was used to processed raw data for both format conversion and de-multiplexing. The Fastq files have been deposited at SRA database under the accession: SRP078773.

### *C*. *rota* transcriptome assembly and data analysis

To assemble the *C*. *rota* transcriptome all 24 of the sampled time points were used. The Illumina sequencing analysis provided a total of 311,558,569 raw sequencing reads. The different time points sampled ranged in size from approximately 6 to 33 million reads. Trim Galore software (www. bioinformatics.babraham.ac.uk/projects/trim_galore/) was used for trimming off sequencing adapters, low quality reads and for quality control. After trimming and QC we remained with a total of 108,167,257 paired-end high quality reads which were used for the Trinity assembly [version r2031110]^64^. The reads were submitted to Trinity software (www.trinityrnaseq.sourceforge.net) for *de novo* assembly using the default parameters. RSEM^65^ was then applied to estimate expression values (RPKM). This stage was used in order to filter out low supported transcripts and to minimize false positive isoforms (IsoPct > 1%). After filtering for isorfoms and low quality reads, the de novo assembly yielded 114,397 (238,126 before RSEM filtering) comprised of 156,322,855 base pairs. Contigs with an average length of 1,366 ± 1,707 bp. Half of the contigs (N50) were 2,762 bp long. Average GC composition was 34.63%. Putative coding regions were extracted from the transcripts using TransDecoder (www.transdecoder.sourceforge.net), providing all the CDS and proteins from the assembly. TransDecoder identified 38,482 (71,104 before initial filtering) putative ORF contigs. Bowtie (v2.1.0) was used to map all the clean high quality reads, normalizing and calculating the expression values. As there is no reference genome for *C*. *rota*, we used NCBI-Blast to reference the contigs from the assembly against the Uniref50 and *Homo sapiens* databses (NCBI), respectively. After filtering both blast results at a cutoff of P > 10*^05^ we remained with a total of 14,434 Uniref and 13,486 *H*. *sapiens* genes with genomic positions which were matched to the *C*. *rota* transcriptome. We applied additional stringent filtering on the blastp results in order to increase the certainty that true *H*. *sapiens* homologs were received (for IPA pathway analysis, see below).

### Rhythmicity analysis

Transcript expression rhythmicity was performed using JTK_Cycle [v2.1]^30^. JTK_Cycle is a statistical non-parametric based algorithm designed to efficiently identify and characterize cycling variables in large data sets. We used the aforementioned transcript list with expression values for each of the samples time points. We conducted two separate analyses of the data. The first analysis was performed in order to detect tidally oscillating transcripts. The data collections of the two 48 hour samplings were considered to be biological replicates. They were staggered to align tidal phase change between sampling weeks. Thus from 24 time points (12 per sampling) the rhythmicity was computed for 13 total time points, 2 replicates in the middle, and one at each end. The parameters were set at detection of 12 hour oscillations. For the diel analysis the two samplings were considered as biological replicates and averaged, resulting in 12 time points. The parameters were set at detection of 24 hour oscillations. For both the tidal and diel analyses we only looked for transcripts with a precise 12 or 24 hour rhythm, and not a range (e.g. 10–14 or 20–28 hours) as is commonplace. This was done primarily to further ensure a small but high confidence data set. A total of 38,482 transcripts were applied to the analysis, including transcripts with no or low confidence annotation. For both analyses only transcripts with an expression median > 0 were used. Amplitude confidence level set at 95%. For both analyses we assessed the number of significant cycling transcripts, taking into account the false detection ratio (q values; Table S2 and Table S3). All transcripts with a resulting p < 0.005 (corresponding to q ≥ 0.48 for the tidal and q ≥ 0.78 for the diel analyses) were considered as confident cyclers. The FDR is crucial when dealing with large genomic experiments, and the choice of threshold is experiment dependent^66^. For the purpose of investigating genes of interest that arose from the analyses were retained the stringent filtering methods mentioned above, however for the purpose of creating heat maps we did not use these measures as the transcripts with a low confidence annotation are presumably *bona fide* cycling transcripts unique to the *C*. *rota* proteome with little or no similarity to the *H*. *sapiens* or Uniprot databases. Heat maps were generated using Partek Genomics Suite software (version 6.6, Copyright©2012, Partek Inc., St. Louis, MO, USA).

### Pathway analysis

Pathway analysis of annotated sequences was performed using the IPA software (http://www.ingenuity.com). The dynamic canonical pathways contained in IPA are well characterized metabolic and cell-signaling pathways that are compiled from the literature and the Kyoto encyclopedia of genes and genomes (KEGG). The IPA canonical pathways display genes/proteins involved, their interactions and the cellular and metabolic reactions in which the pathway is involved. Since the focus of this study is rhythmicity, we conducted two analyses, using the significant cycling gene lists identified by JTK_Cycle (P < 0.005). In addition we further filtered the gene list in order to assure the validity of the *Homo sapiens* annotations, removing those with a low confidence sequence identity and coverage, resulting in 260 tidal and 150 diel cyclers, respectively. The analysis was run against the curated IPA database using default settings. Expression values were z-score normalized and a comparison was conducted in order to identify enriched pathways with a significant differential expression between the sampled time points (13 tidal and 12 diel, as above). We further noted IPA’s predicted upstream regulators. Resulting enriched pathways and predicted upstream regulators were plotted in heatmaps using Partek Genomics Suite software (version 6.6, Copyright©2012, Partek Inc., St. Louis, MO, USA). All IPA results were filtered with a P < 0.05.

### Validations - Droplet Digital PCR (ddPCR)

Specific *timeless* and *cry1*, *period* and *beta- actin* (housekeeping) primers and probe were planned and sequenced by Agentek (www.agentek.co.il; Table S5). The primers were first verified with rt-PCR and agarose gel. Subsequently gradient PCR was used to establish the optimal annealing temperature. cDNA was quantified using the QX100TM Droplet DigitalTM PCR system (Bio-Rad, Pleasanton, CA). The ddPCR mix consisted of: 10 ml 2× ddPCRTM super mix for probes (Bio-Rad); 200 nM of forward and reverse primers; 400 nM probe mix and 4 ml of the cDNA into a final volume of 20 ml. The total mix was placed into an 8 channel cartridge, 50 ml of droplet generating oil was added and droplets were formed in the QX100TM droplet generator (Bio-Rad). Droplets in oil suspensions were transferred to an Eppendorf 96 well plate (Eppendorf, Germany) and placed into the T100TM Thermal Cycler (Bio-Rad). Cycling conditions were as follows: 95 °C for 5 min, followed by 40 cycles of 95 °C for 15 sec and 58 °C for 60 sec. The droplets were subsequently read automatically by the QX100TM droplet reader (Bio-Rad) and the data was analyzed with the QuantaSoftTM analysis software 1.3.2.0 (Bio-Rad), and then exported to Excel (Microsoft) for further analysis.

### Validations - Quantitative real time polymerase chain reaction (qPCR)

Specific *rhodopsin* (target gene) and *Importin 3* (reference gene) primers were planned with the IDT primer quest function (Table S5). *Importin 3* exhibited appropriate levels of expression in the transcriptome data and was later verified with qPCR. Both primer sets were sequenced by IDT. The primers were first verified with rt-PCR and agarose gel. The primers were then checked for efficiency using a 5 fold dilution series (1:4, 1:16, 1:64, 1:256, and 1:1024). The standard curve was used in the qPCR reactions to quantify amplification efficiency. The efficiency of both genes was over 90%. The qPCR reaction master-mix was prepared as follows: 5 μl GoTaq® qPCR Master Mix (Promega), 0.25 μl of each primer (diluted 1:10), 4 μl of 100× diluted cDNA and 0.5 μl nuclease free water. qPCR reactions were loaded in triplicate and assays were performed following the program proposed by GoTaq® qPCR Master Mix protocol. The qPCR data were then transformed and normalized according to the delta-delta Ct model^67^. The planning and sequencing of primers was conducted as above. Reactions were performed on a Corbett research Rotor-Gene 6000.

### Statistical analyses

For all the behavioural data, collected in the sea and laboratory, rhythmicity was calculated using the LSP program (written by R. Refinetti; http://www.circadian.org/softwar.html) which employs a Lomb-Scargle based algorithm. The Lomb-Scargle periodogram analysis is the most suited for our data and used widely in similar studies (Pers. comm. R. Refinetti;^68–71^). For the limpet locomotion analysis nested ANOVA was used to test for the effect of tidal phase, season and moon phase for each of the different periods quantified. The data was rank transformed for this purpose. This was followed by a Tukey post-hoc test for each season and lunar phase. Non-parametric Man-Whitney U and Kruskall-Wallis tests were applied when relevant. Difference between lag in locomotion during the spray experiment was conducted with a t-test. In the molecular analysis, JTK_Cycle statistical methods are presented in the JTK_Cycle section above. Chi-square test was employed to evaluate the difference between tidal cyclers peaking at high vs. low tide, as well as diel cyclers peaking at midday vs. midnight. Fisher’s exact test was used in IPA analysis to estimate the significance of the incidence of different canonical pathways. This method calculates the probability that the association between experimental gene set and the reference gene set associated with a canonical pathway is due to random chance. A p-value < 0.05 was considered statistically significant and indicated a nonrandom enrichment of an experimental dataset by members of a specific pathway.

## Electronic supplementary material

Movie S1

Supplementary Information

File S1

File S2

File S3

## Data Availability

Raw transcriptome read data has been deposited into the NCBI Short Read Archive (SRA) under accession number SRP078773. The sequences used for C. rota identification have been deposited into the GenBank database under the accessions: KX453271-KX453283.
