# Publisher Correction: Tidal and diel orchestration of behaviour and gene expression in an intertidal mollusc

**DOI:** 10.1038/s41598-019-46787-4

**Published:** 2019-07-17

**Authors:** Y. Schnytzer, N. Simon-Blecher, J. Li, H. Waldman Ben-Asher, M. Salmon-Divon, Y. Achituv, M. E. Hughes, O. Levy

**Affiliations:** 1 The Mina and Everard Goodman Faculty of Life Sciences, Bar-Ilan University, Ramat-Gan, Israel; 2 Present Address: Eugene Bell Center for Regenerative Biology and Tissue Engineering, Marine Biological Laboratory, Woods Hole, MA USA; 3 Division of Pulmonary and Critical Care Medicine, Washington University School of Medicine, St. Louis, MO USA; 4 Department of Molecular Biology, Ariel University, Ariel, Israel

Correction to: *Scientific Reports* 10.1038/s41598-018-23167-y, published online 20 March 2018

In the original version of this Article, the author H. Waldman Ben-Asher was incorrectly indexed. This error has now been corrected.

