# Supplementary material for: Tidal and diel orchestration of behaviour and gene expression in an intertidal mollusc
Source: Sci Rep. 2018 Mar 20;8:4917. doi: 10.1038/s41598-018-23167-y (PMC5861051; doi:10.1038/s41598-018-23167-y)
Supplement: Supplementary file 2 — Supplementary Information [file 41598_2018_23167_MOESM2_ESM.docx]

**Supplementary information**

**Tidal and diel orchestration of behaviour and gene expression in an intertidal mollusc**

Y. Schnytzer^1*^, N. Simon-Blecher^1^, J. Li^2^, H. Waldman Ben-Asher^1^, M. Salmon-Divon^3^, Y. Achituv^1^, M.E. Hughes^2^, O. Levy^1*^

^1^Bar-Ilan University, The Mina and Everard Goodman Faculty of Life Sciences, Ramat-Gan, Israel; ^2^University of Missouri-St. Louis, Department of Biology, MO, USA; ^3^Ariel University, Department of Molecular Biology, Ariel, Israel.

Corresponding authors email: [yschnytzer@mbl.edu](mailto:yschnytzer@mbl.edu) & [oren.levy@biu.ac.il](mailto:oren.levy@biu.ac.il)

**Supplemental figures and tables**

**
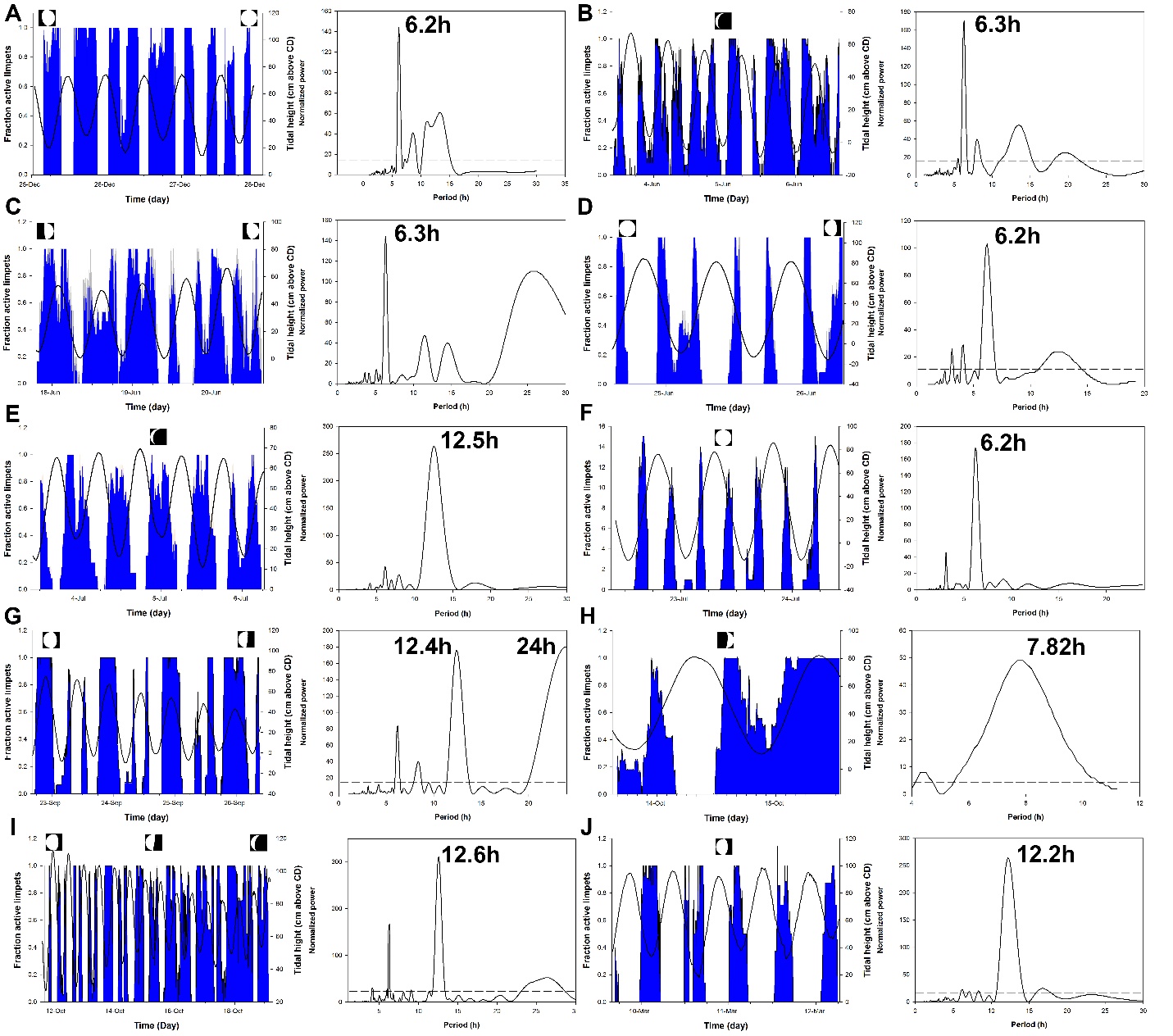
**

**Figure S1.** **Locomotor activity of *C. rota* under natural conditions in the field over the course of the study**. Presented periods - (A) 25/12/2012-28/12/2012 (n=16), (B) 3/6/2013-6/6/2013 (n=24), (C) 17/6/2013-20/6/2013 (n=19), (D) 24/6/2013-26/6/2013 (n=17), (E) 3/7/2013-6/7/2013 (n=18), (F) 22/7/2013-24/7/2013 (n=18), (G) 22/9/2013-26/9/2013 (n=17), (H) 14/10/2013-15/10/2014 (n=17), (I) 11/10/2014-19/10/2014 (n=97), (J) 9/3/2015-12/3/2015 (n=20). The blue filled plot represents the fraction of active limpets. The black line plot represents the sea level at the time. X-axis is time (days), primary y-axis is fraction of active limpets, and secondary y-axis is sea level. Moon phase is presented above the graph. Numbers of limpets monitored during each time period are noted above (max observed during given period). LSP periodograms are presented to the right of each activity graph. Most significant peaks are labeled (p<0.05). Dashed line in the periodograms indicated the 95% confidence intervals.

**Table S1.** **List of periods photographed and analyzed in Eilat at the sea observation location.**

**
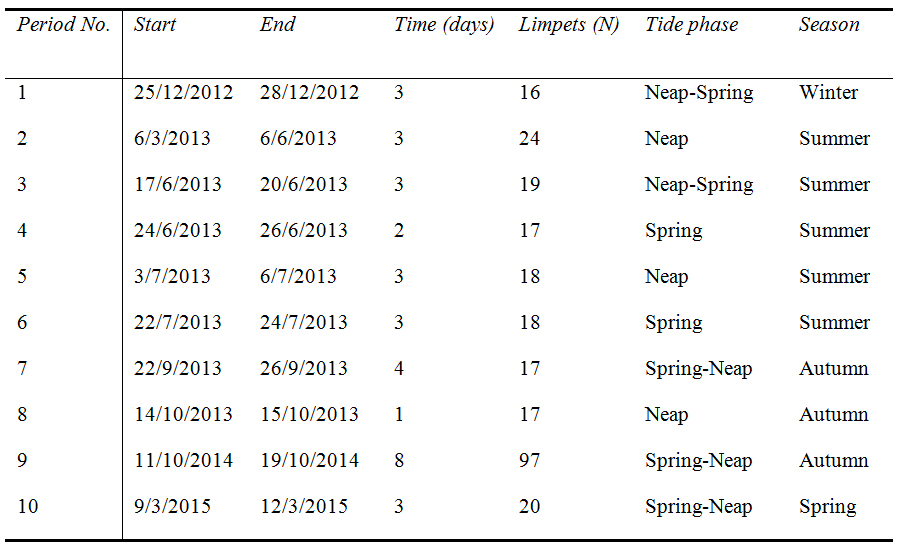
**

**Supplemental Movie – Timelapse video of *Cellana rota* movement.** Video composed of one image taken every 5 minutes during the day and night (IR illumination) over the course of three days (corresponds to Panel B in figure S1 and Period 2 in Table S1). Speed x16 for viewer convenience. The limpets can be seen moving up and down the boulder with the rising and falling of the tide. No movement during high and low tides.

**
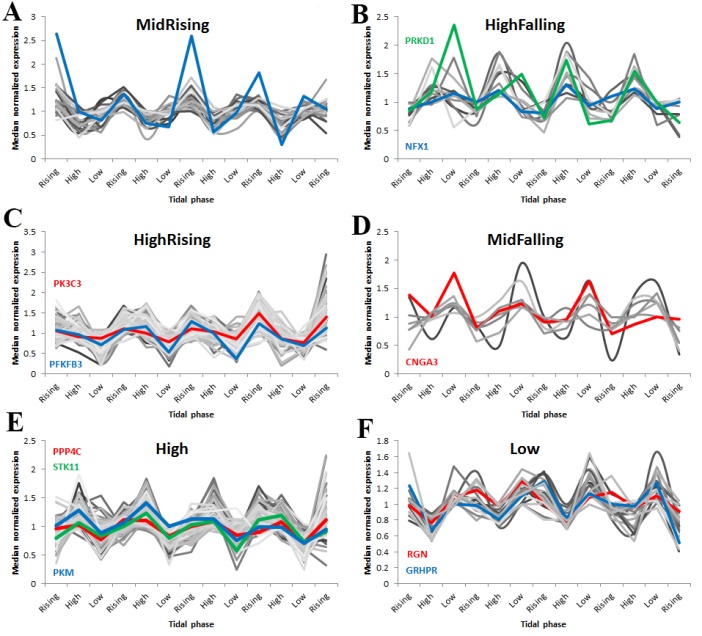
**

**Figure S2. Rhythmic expression of tidal cyclers.** Phases are sorted by the lag values given by JTK_Cycle. Representative circadian clock related genes marked in red or green, hypoxia /anoxia related in blue.

**Table S2. Tidally rhythmic transcripts identified with JTK_Cycle.**

**
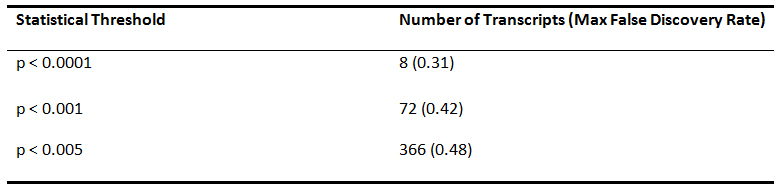
**

**
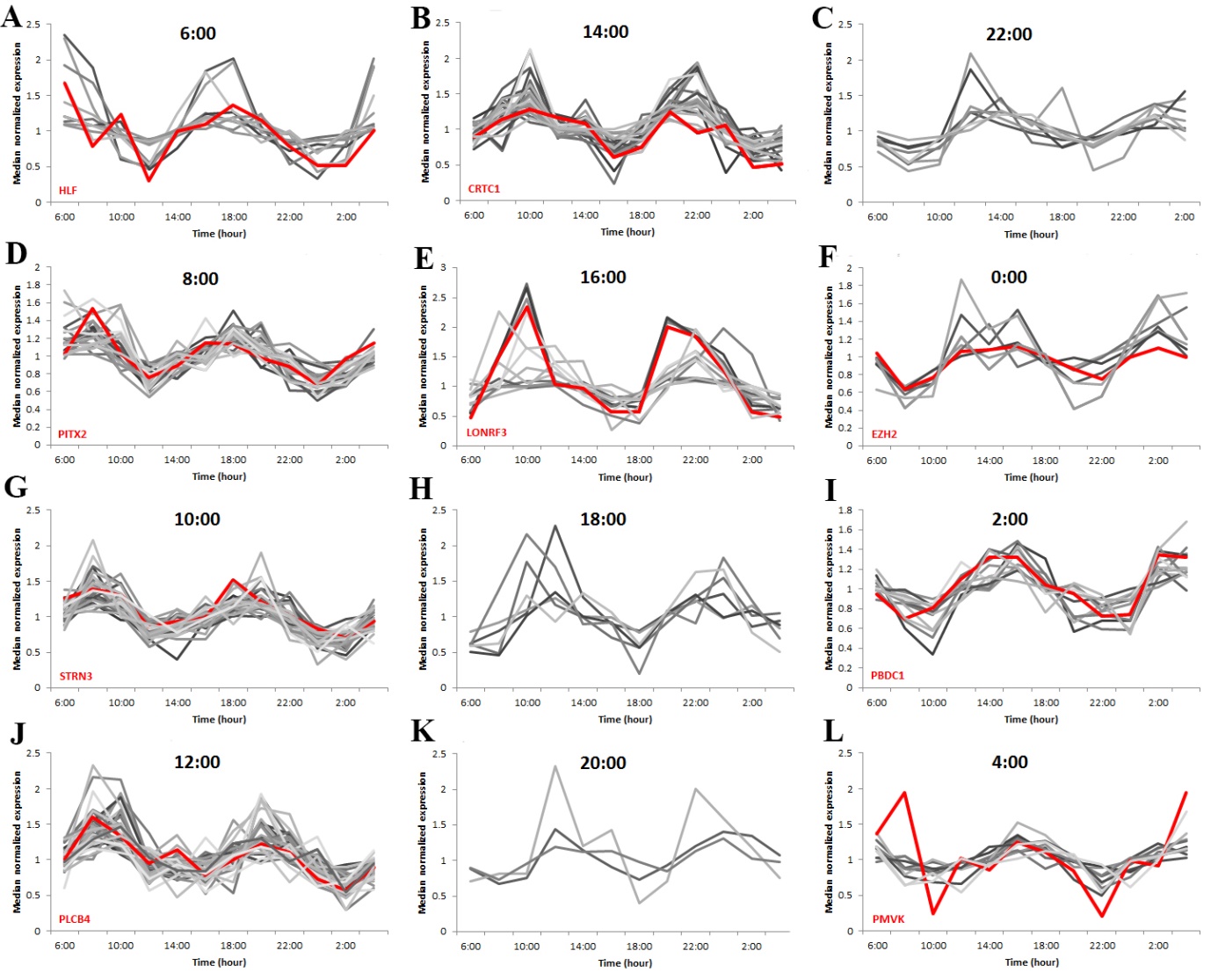
**

**Figure S3. Rhythmic expression of diel cyclers.** Phases are sorted by the lag values given by JTK_Cycle. Representative circadian clock related genes marked in red.

**Table S3. Diel rhythmic transcripts identified with JTK_Cycle.**

**
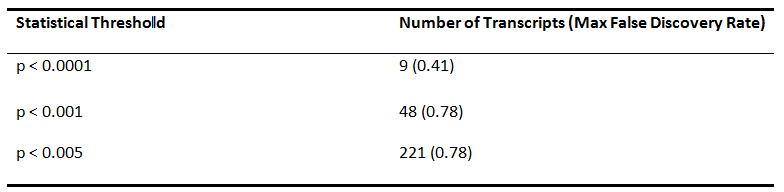
**

**Table S4.** **Top tidally cycling transcripts of interest.**

**
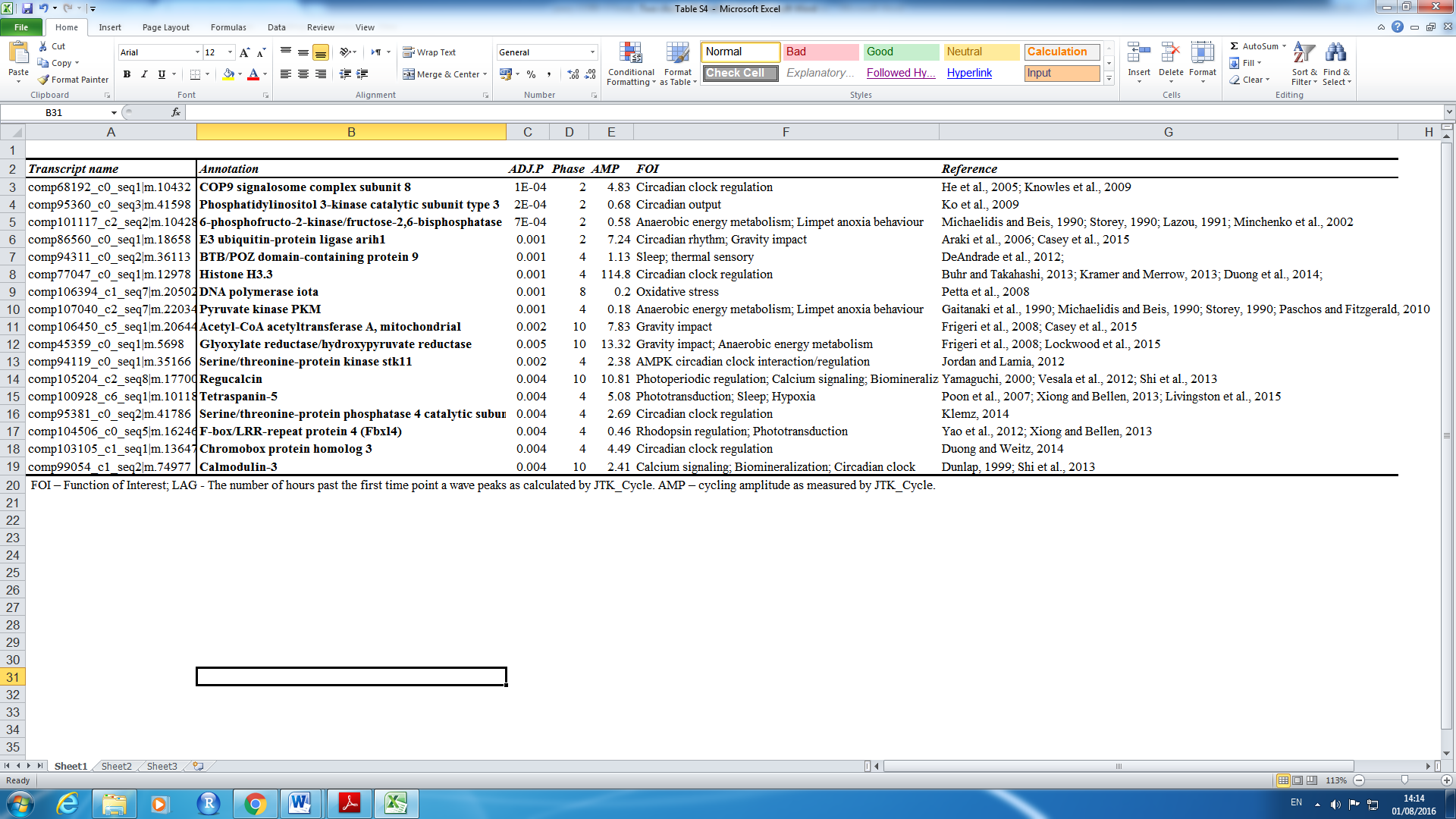
**

**
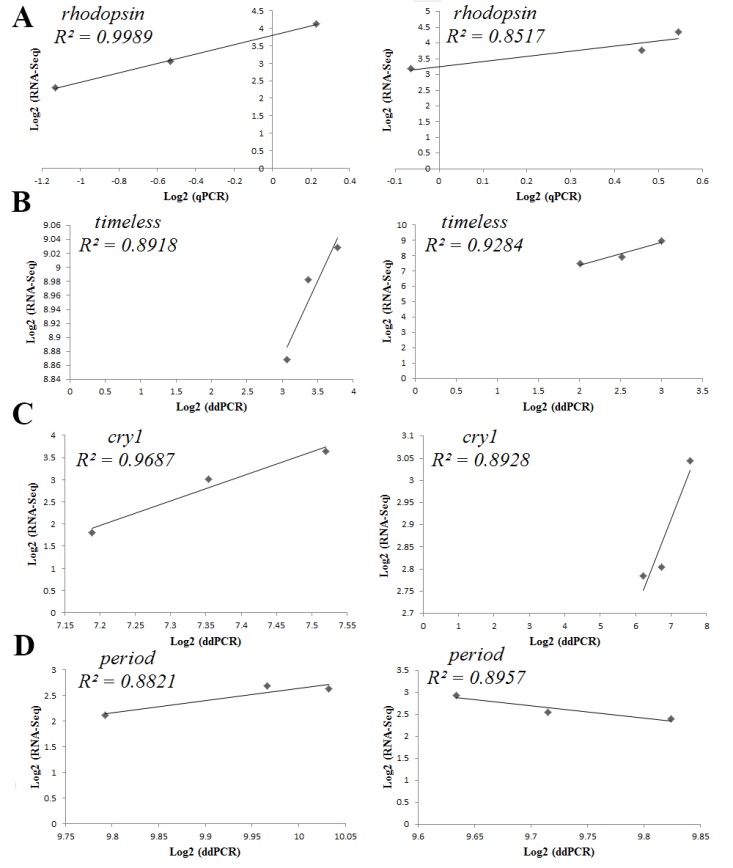
**

**Figure S4. Correlation of gene expression (Log2) between values obtained from RNA-seq and Real-Time PCR (qPCR and ddPCR) analyses.** Four genes were chosen for validation. For each of the genes six time points were chosen (10:00, 02:00 and 10:00 X two sampling weeks). Genes and quantification method used:  *rhodopsin* was tested using qPCR with *importin3* as a reference gene. *timeless*, *cry1* and *period* were tested using ddPCR, *beta-actin* used as a reference gene. N=5 for each sample. Left column is Week1 sampling (25/12/2012-27/12/2012), and right column is week2 sampling (1/1/2013-3/1/2013).


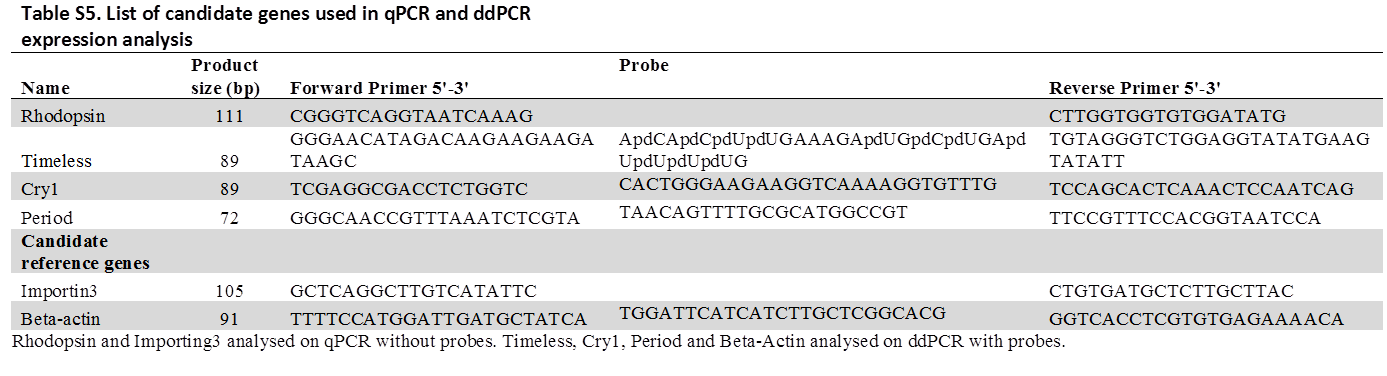


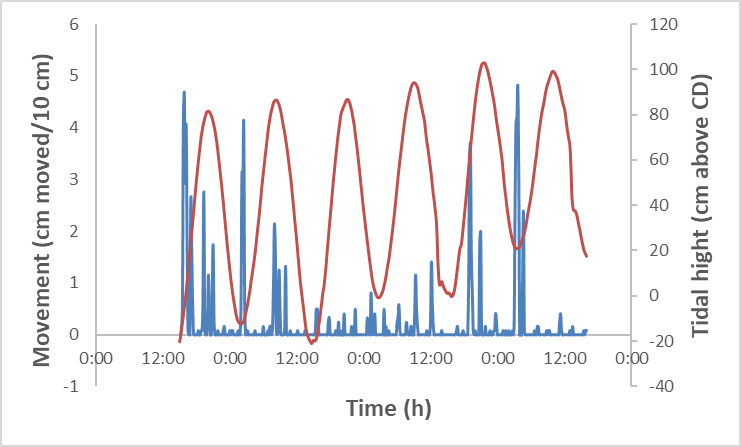


**Figure S5. *Cellana rota* behaviour in the laboratory.** Example of limpet under LD exhibiting atypical ~8 h rhythmicity (72 hours; no tide; τ=8.2). Tidal phase at collection location plotted in red. Locomotion plotted in blue. Arrows denote time of spray turning on for a period of 3 hours each time (spaced 12.4 hours apart). Period calculated with LSP periodogram (p>0.05).
